# Supplementary material for: Selective loss of CD107a TIGIT+ memory HIV-1-specific CD8+ T cells in PLWH over a decade of ART
Source: eLife. 2023 Sep 19;12:e83737. doi: 10.7554/eLife.83737 (PMC10508883; doi:10.7554/eLife.83737)
Supplement: Supplementary file 1. [file elife-83737-supp1.docx]

| **Supplementary file 1 - Table 1.** | | | | |  |
| --- | --- | --- | --- | --- | --- |
| Study Groups | **Healthy Control** | **Early HIV-1 infection** | **HIV-1 on ART** | | ƥ |
|  | HC | Ei | S | |  |
| Number of subjects | 24 | 24 | 24 | |  |
| Gender (M/F)  [n (%)] | 16/8  (67/33%) | 23/1  (96/4%) | 18/6  (75/25%) | | 0.091 |
| Weeks since seroconversion  [median (IQR)] | - | 1.3  (0.77 - 17.8) | - | - |  |
| Number of samples | 24 | 24 | 24 (S1) | 24 (S2) |  |
| Age at sample (years)  [median (IQR)] | 43.1  (38.1 - 52.5) | 43.3  (41.8 - 46.2) | 40.6  (35.1 - 44.7) | 50.0  (43.7 - 55.1) | 0.0035 |
|  |  |  |  | |  |
| CD4+ T-cell counts at sample per mm^3^  [median (IQR)] | - | 630  (557 - 757.3) | 573  (464 - 992) | 910  (799.8 - 1111) | 0.0001 |
|  |  |  |  |  |  |
| Years undetectable at sample on ART [median (IQR)] | - | 0.0 | 2.2  (1.8 - 2.8) | 10.1  (7.4 - 12.9) | <0.0001 |
| VL log copies/ml at sample [median (IQR)] | - | 4.5 (4.3 - 5.1) | undetectable | undetectable | <0.0001 |
| Abbreviations: IQR, interquartile range; P-values were calculated using Kruskal-Wallis test (Dunn´s correction) or X^2^-test. | | | | | |

**The epidemiological and clinical characteristics of the study groups.**

Table showing the clinical data and biological sample availability from study groups, healthy controls (HC), PLWH in early HIV-1 infection (Ei), and PLWH on fully suppressive ART (S) in S1 and S2 time points. Ei individuals were defined in a window of 1.3 (0.77 - 17.8) weeks after seroconversion in the absence of ART. PLWH on fully suppressive ART (S) in S1 and S2 time points were treated mainly with a combination of NNRTI and NRTI for more than ten years with sustained virological suppression (<50 HIV-1-RNA copies/ml). We excluded individuals with integrase inhibitors, ART as monotherapy, and treatments with mitochondrial toxicity, including Trizivir, d4T, ddI, AZT and blips over the ART period (S1- S2) to ensure homogeneous treatment over time. All groups were balanced by age to the S2 samples to avoid confounding effects on IR expression.
